# Supplementary material for: ZZ-dependent regulation of p62/SQSTM1 in autophagy
Source: Nat Commun. 2018 Oct 22;9:4373. doi: 10.1038/s41467-018-06878-8 (PMC6197226; doi:10.1038/s41467-018-06878-8)
Supplement: Supplementary file 1 — Supplementary Information [file 41467_2018_6878_MOESM1_ESM.pdf]

**Supplementary Information**

# ZZ-Dependent regulation of p62/SQSTM1 in autophagy

Yi Zhang, Su Ran Mun, et al.,

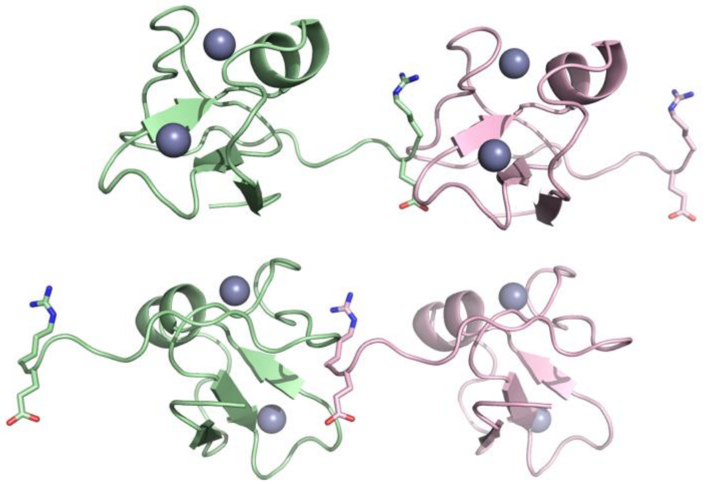

**Supplementary Figure 1.** The crystal structure of the RELGS-ZZ construct.

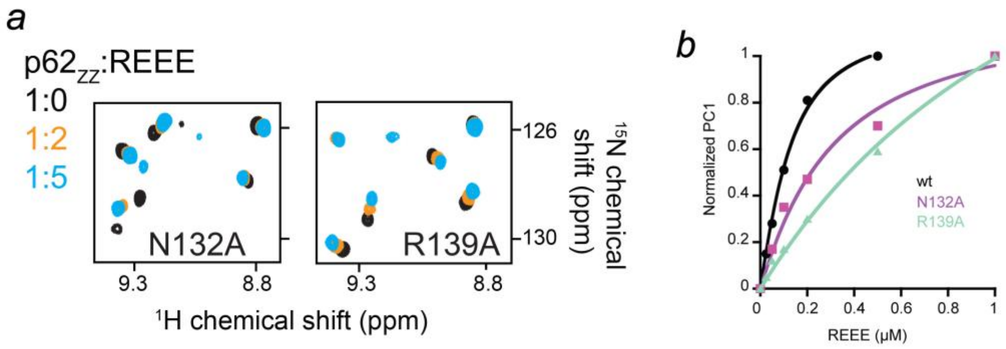


**Supplementary Figure 2.** (a) Superimposed $^{1}H$,$^{15}N$ HSQC spectra of the indicated mutants of $p62_{ZZ\ (115\text{-}190)}$ collected upon titration with the REEE peptide. Spectra are color coded according to the protein:peptide molar ratio. (b) Binding isotherms for the interaction of WT $p62_{ZZ\ (115\text{-}190)}$ and N132A and R139A mutants with the REEE peptide obtained using NMR principal component analysis (PCA) implemented with TREND software[1].

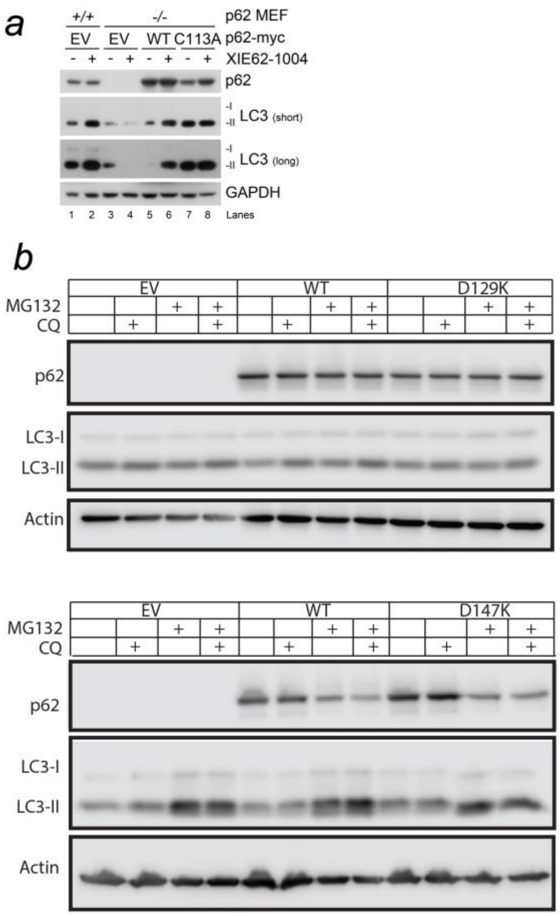


**Supplementary Figure 3.** (a) p62 aggregation via Cys113 is also essential for induction of autophagy with XIE62-1004. Treatment of p62 +/+ cells with XIE62-1004 induced LC3-II lipidation, whereas p62 -/- cells were non-responsive. As expected, transient expression of recombinant p62-myc in p62 -/- MEFs rescued their ability to induce LC3-II lipidation upon XIE62-1004 treatment. Importantly, p62 C113A mutant failed to induce autophagy upon XIE62-1004 treatment. Intriguingly, the mere expression of the p62 C113A mutant was sufficient to increase the level of LC3-II. (b) Western blot for LC3 I/II, p62 and β-Actin on whole cell lysates harvested from p62-/- MEFs with stable expression of an empty vector, WT p62, D129K p62 (top) or D147K (bottom) p62 after treatment with MG132 (500nM) or CQ for 24hrs.

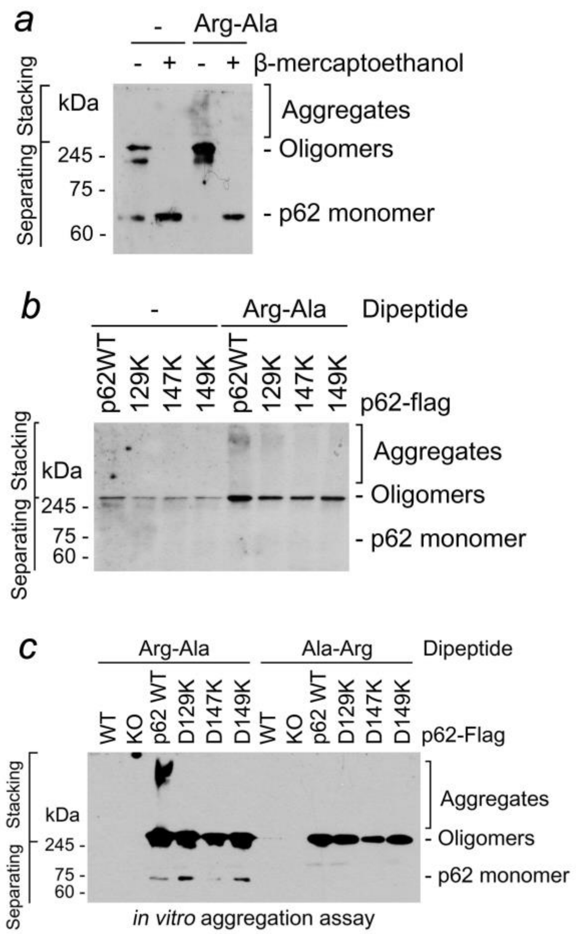


**Supplementary Figure 4.** (a) *In vitro* aggregation assays of wild-type p62 stably expressed in p62 f/f MEFs with or without β-mercaptoethanol. (b) *In vitro* aggregation assays of wild-type or mutated p62 stably expressed in p62 f/f MEFs treated with or without Arg-Ala. (c) *In vitro* aggregation assays of wild-type or mutated p62 stably expressed in p62 f/f MEFs treated with Arg-Ala or Ala-Arg.

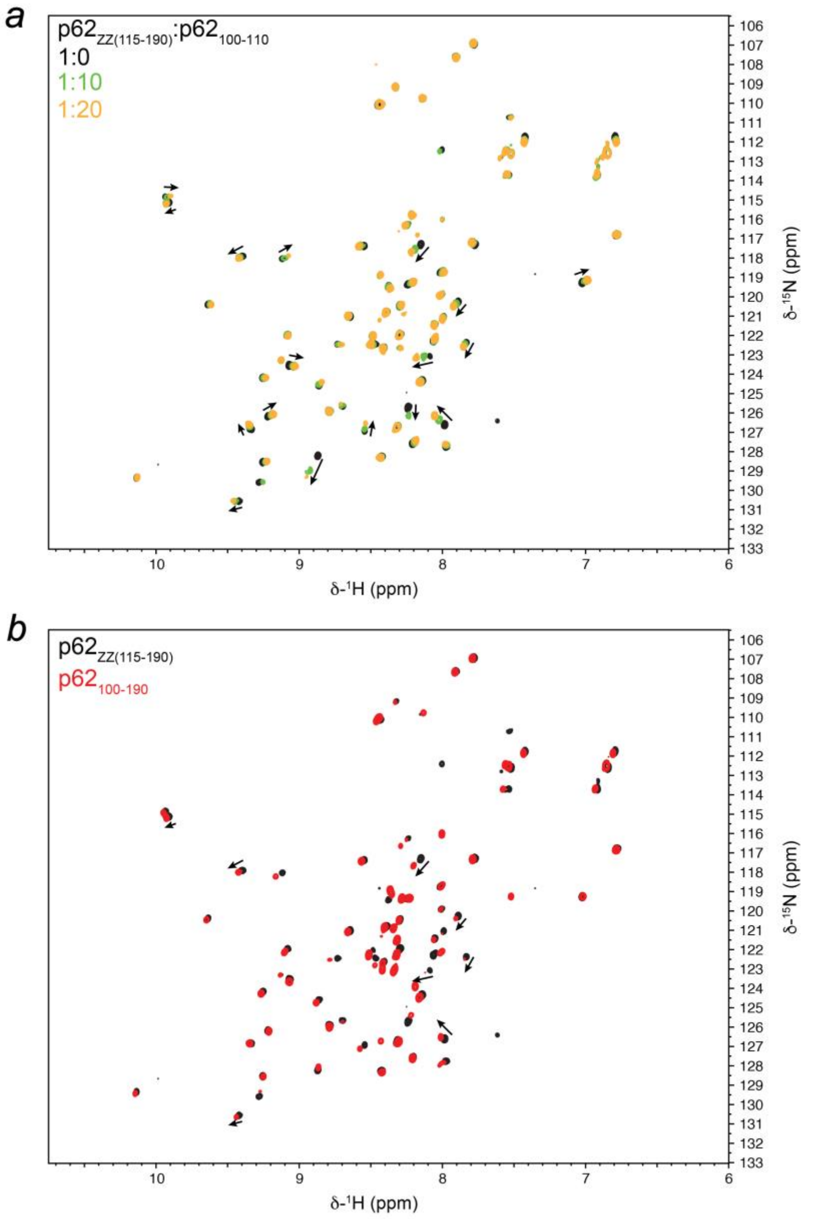


**Supplementary Figure 5.** (a) Superimposed $^{1}$H,$^{15}$N HSQC spectra of p62$_{ZZ\ (115\text{-}190)}$ collected upon titration with the p62$_{ZZ\ (100\text{-}110)}$ peptide. Spectra are color coded according to the protein:peptide molar ratio. (b) Superimposed $^{1}$H,$^{15}$N HSQC spectra of p62$_{ZZ\ (115\text{-}190)}$ and p62$_{RL\text{-}ZZ\ (100\text{-}190)}$ in apo-states.

**Primer Sequences**

**p62 D129K**
5'-ctgcaaaggctgcaatgggcctgtggtaggaac-3'
5'-aacatggtgcaccccaatgtgatctgcaaaggc-3'

**p62 D147K**
5'-gcccaaaatacgacttgtgtagcgtctgcgaggg-3'
5'-cccgctacaagtgcagcgtctgcccaaaatac-3'

**p62 D149K**
5'-ctacaaattgtgtagcgtctgcgagggaaagggc-3'
5'-cccgctacaagtgcagcgtctgcccagactacaaattg-3'

**p62 115-190**
5'-ccctgggatcccaggaggcgccccgcaacatggtg-3'
5'-gttctgttccaggggcccctgggatcccaggaggcg-3'

**p62 120-190**
5'-ccctgggatccaacatggtgcaccccaatgtgatctgcgatggc-3'
5'-gttctgttccaggggcccctgggatccaacatggtgcacc-3'

**NdeI_pCIOX_F**
5'-AGATATACATATGGGCAGCAG-3'

**p62_171_EcoRI_R**
5'-GAGCTCGAATTCttaGGGGCTGGGGAATGCG-3'

**p62_190_EcoRI_R**
5'-GAGCTCGAATTCttaGTGTTTCACCTTCCGGAG-3'

**Sumo_LGS_p62_120**
5'-GAGAACAGATTGGTGGTctgggtagcAACATGGTGCACCCC-3'
5'-GGGGTGCACCATGTTgctacccagACCACCAATCTGTTCTC-3'

**Sumo_RELGS_p62_120**
5'-GAGAACAGATTGGTGGTcgtgagctgggtagcAACATGGTGCACCCC-3'
5'-GGGGTGCACCATGTTgctacccagctcacgACCACCAATCTGTTCTC-3'

**Supplementary Figure 6.** List of primers.

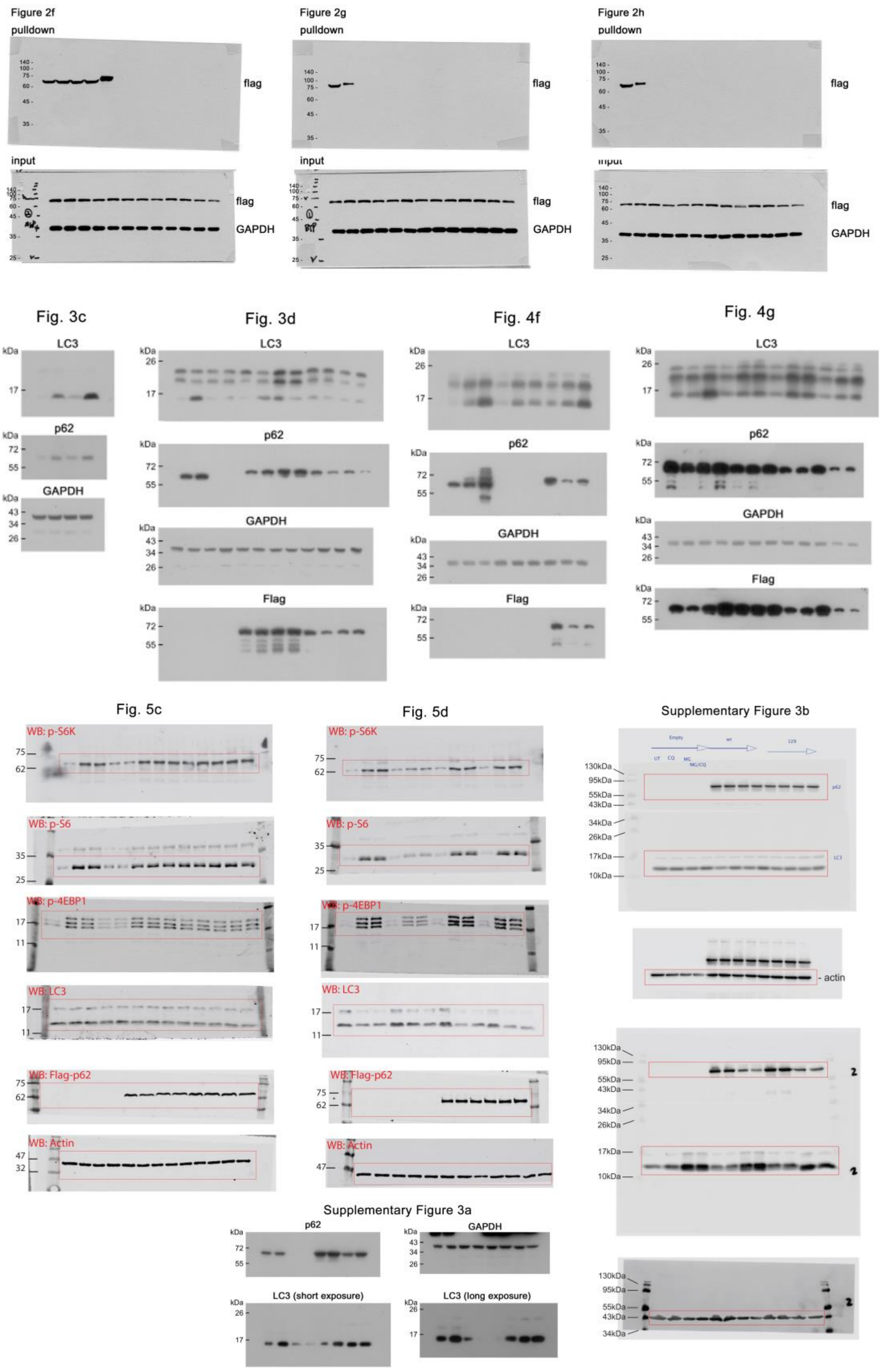


**Supplementary Figure 7.** Uncropped Western blots.

**Supplementary Table 1.** Data collection and refinement statistics for the $p62_{ZZ}$ complexes

| | RE-ZZ | ZZ/Arginine |
|---|---|---|
| **Data collection** | | |
| Wavelength | 1.5418 Å | 1.2782 Å |
| Space group | P1 | C2 |
| Resolution (Å) | 25.31–1.90 | 50.00-1.41 |
| Cell dimensions (Å) | | |
| | a=26.99 | a=44.97 |
| | b=27.86 | b=32.27 |
| | c=38.99 | c=34.94 |
| | **α**=90.06° | **α**=**γ**=90.00° |
| | **β**=90.45° | **β**=122.16° |
| | **γ**=110.3° | |
| Number of measured reflections | 51427 | 107631 |
| Number of unique reflections | 8404 | 8142 |
| Completeness(%) | 99.5 (93.1) | 98.4 (78.4) |
| Redundancy | 6.1 (3.0) | 13.2 (9.3) |
| I/sigma (I) | 27.8 (5.1) | 83.2 (45.5) |
| Rmerge(%) | 6.4 (19.9) | 6.3 (16.5) |
| **Refinement** | | |
| Resolution (Å) | 25.31-1.90 | 29.58-1.41 |
| Number of reflections | 14195 | 8142 |
| R-factor (%) | 0.1546 | 0.1356 |
| R-free (%) | 0.2123 | 0.1646 |
| Number of protein atoms | 888 | 422 |
| Number of Arginine | - | 12 |
| Number of Zinc | 4 | 2 |
| Number of 1,4-Dioxane | - | 6 |
| Number of water molecules | 133 | 107 |
| R.m.s.d. from ideal values | | |
| Bond lengths (Å) | 0.007 | 0.005 |
| Bond angles (°) | 1.080 | 0.82 |
| Average B-values (Å$^2$) | 25.8 | 13.3 |
| Protein chain A | 20.2 | 9.9 |
| Protein chain B | 19.2 | |
| Water | 31.2 | 26.9 |
| Zinc | 19.3 | 8.8 |
| Arginine | - | 15.4 |
| 1,4-Dioxane | - | 12.7 |
| Ramachandran Plot analysis | | |
| Favored | 95.6% | 96.2% |
| Allowed | 4.4% | 3.9% |
| Outliers | 0% | 0% |

* Values in parentheses refer to data in the highest resolution shell. Datasets collected from a single crystal.

**Supplementary References:**

1. Xu, J. & Van Doren, S.R. Tracking Equilibrium and Nonequilibrium Shifts in Data with TREND. *Biophys J* **112**, 224-233 (2017).

**Supplementary Data 1.** List of antibodies.
