# Supplementary material for: ZZ-dependent regulation of p62/SQSTM1 in autophagy
Source: Nat Commun. 2018 Oct 22;9:4373. doi: 10.1038/s41467-018-06878-8 (PMC6197226; doi:10.1038/s41467-018-06878-8)
Supplement: Supplementary file 3 — Description of Additional Supplementary Files [file 41467_2018_6878_MOESM3_ESM.pdf]

Description of Additional Supplementary Files

File Name: Supplementary Data 1
Description: List of antibodies.
