# Supplementary material for: ZZ-dependent regulation of p62/SQSTM1 in autophagy
Source: Nat Commun. 2018 Oct 22;9:4373. doi: 10.1038/s41467-018-06878-8 (PMC6197226; doi:10.1038/s41467-018-06878-8)
Supplement: Supplementary file 5 — Reporting Summary [file 41467_2018_6878_MOESM5_ESM.pdf]

nature research

Corresponding author(s): *Double-blind peer review submissions: write DBPR and your manuscript number here instead of author names.*

# Reporting Summary

Nature Research wishes to improve the reproducibility of the work that we publish. This form provides structure for consistency and transparency in reporting. For further information on Nature Research policies, see Authors & Referees and the Editorial Policy Checklist.

## Statistical parameters

When statistical analyses are reported, confirm that the following items are present in the relevant location (e.g. figure legend, table legend, main text, or Methods section).

| n/a | Confirmed | |
|---|---|---|
| ☐ | ☒ | The exact sample size (*n*) for each experimental group/condition, given as a discrete number and unit of measurement |
| ☐ | ☒ | An indication of whether measurements were taken from distinct samples or whether the same sample was measured repeatedly |
| ☐ | ☒ | The statistical test(s) used AND whether they are one- or two-sided<br>*Only common tests should be described solely by name; describe more complex techniques in the Methods section.* |
| ☒ | ☐ | A description of all covariates tested |
| ☒ | ☐ | A description of any assumptions or corrections, such as tests of normality and adjustment for multiple comparisons |
| ☒ | ☐ | A full description of the statistics including central tendency (e.g. means) or other basic estimates (e.g. regression coefficient) AND variation (e.g. standard deviation) or associated estimates of uncertainty (e.g. confidence intervals) |
| ☒ | ☐ | For null hypothesis testing, the test statistic (e.g. *F*, *t*, *r*) with confidence intervals, effect sizes, degrees of freedom and *P* value noted<br>*Give P values as exact values whenever suitable.* |
| ☒ | ☐ | For Bayesian analysis, information on the choice of priors and Markov chain Monte Carlo settings |
| ☒ | ☐ | For hierarchical and complex designs, identification of the appropriate level for tests and full reporting of outcomes |
| ☒ | ☐ | Estimates of effect sizes (e.g. Cohen's *d*, Pearson's *r*), indicating how they were calculated |
| ☐ | ☒ | Clearly defined error bars<br>*State explicitly what error bars represent (e.g. SD, SE, CI)* |

*Our web collection on statistics for biologists may be useful.*

## Software and code

Policy information about availability of computer code

| | |
|---|---|
| Data collection | X-ray crystallographic data were collected at beam line 4.2.2 of the ALS in Berkeley and on a Rigaku Micromax 007 high-frequency microfocus X-ray generator at the UC Denver Biophysical Core facility |
| Data analysis | PHENIX, Coot, and other software listed in the Method section. |

For manuscripts utilizing custom algorithms or software that are central to the research but not yet described in published literature, software must be made available to editors/reviewers upon request. We strongly encourage code deposition in a community repository (e.g. GitHub). See the Nature Research guidelines for submitting code & software for further information.

## Data

Policy information about availability of data

All manuscripts must include a data availability statement. This statement should provide the following information, where applicable:

- Accession codes, unique identifiers, or web links for publicly available datasets
- A list of figures that have associated raw data
- A description of any restrictions on data availability

The Coordinates and structure factors have been deposited in the Protein Data Bank under the accession code 6MIU and 6MJ7. Other data are available from the corresponding author upon reasonable request.

# Field-specific reporting

Please select the best fit for your research. If you are not sure, read the appropriate sections before making your selection.

☒ Life sciences ☐ Behavioural & social sciences ☐ Ecological, evolutionary & environmental sciences

For a reference copy of the document with all sections, see nature.com/authors/policies/ReportingSummary-flat.pdf

# Life sciences study design

All studies must disclose on these points even when the disclosure is negative.

| | |
|---|---|
| Sample size | present in relevant figure legends |
| Data exclusions | no data exclusions |
| Replication | present in relevant figure legends |
| Randomization | no randomization |
| Blinding | no blinding |

# Reporting for specific materials, systems and methods

## Materials & experimental systems

| n/a | Involved in the study | |
|---|---|---|
| ☒ | ☐ | Unique biological materials |
| ☐ | ☒ | Antibodies |
| ☐ | ☒ | Eukaryotic cell lines |
| ☒ | ☐ | Palaeontology |
| ☒ | ☐ | Animals and other organisms |
| ☒ | ☐ | Human research participants |

## Methods

| n/a | Involved in the study | |
|---|---|---|
| ☒ | ☐ | ChIP-seq |
| ☐ | ☒ | Flow cytometry |
| ☒ | ☐ | MRI-based neuroimaging |

## Antibodies

| | |
|---|---|
| Antibodies used | The primary antibodies used were: anti-phospho-S6K (Cell Signaling, #9205), anti-phospho-4EBP1 (Cell Signaling, #2855), anti-phospho-S6 (Cell Signaling, #5364), anti-LC3 (Cell Signaling #4108, Sigma #L7543, and Novus #NB100-2220), anti-β-actin (Sigma, #A1978 and #A5441), anti-Flag (Sigma #F3165 and Sigma #F1804), anti-p62 (Novus #H00008878 and Abcam #56416) and anti-GAPDH (BioWorld, #AP0063). The secondary antibodies used were: anti-IgG1 (BD Biosciences #550331, Cell Signaling #7974 and #7076), anti-rabbit (Dako, #E0432) and Alexa fluor488 anti-IgG (Invitrogen #A11034 and #11029). |
| Validation | All antibodies validation are available on the manufacturers' websites. |

## Eukaryotic cell lines

Policy information about cell lines

| | |
|---|---|
| Cell line source(s) | p62-floxed mouse embryo fibroblasts (p62fl/fl MEFs) were derived as previously reported (PMCID: PMC3190169). HEK293 cells were obtained from ATCC. |
| Authentication | p62fl/fl MEFs were not authenticated |
| Mycoplasma contamination | All cell lines were tested negative for mycoplasma contamination |
| Commonly misidentified lines (See ICLAC register) | N/A |

# Flow Cytometry

## Plots

Confirm that:

☒ The axis labels state the marker and fluorochrome used (e.g. CD4-FITC).

☒ The axis scales are clearly visible. Include numbers along axes only for bottom left plot of group (a 'group' is an analysis of identical markers).

☒ All plots are contour plots with outliers or pseudocolor plots.

☒ A numerical value for number of cells or percentage (with statistics) is provided.

## Methodology

| | |
|---|---|
| Sample preparation | p62-/- MEFs and MEFs with stable rescue of WT or Mut p62 constructs were treated as indicated with trypsinized, spun down, and resuspended in Phenol red free medium. |
| Instrument | Gallios 561 (Beckman Coulter) |
| Software | FlowJO |
| Cell population abundance | Figure 4b shows an example of the gated population for cells actively undergoing autophagy after being gated for live cells and single cells and LC3-tandem positivity. |
| Gating strategy | Samples were first gated on live cell cells and single cells based on forward and side scatter. Unstained controls were used to gate for cells stained positive for the LC3-tandem construct. Gating for autophagy is described in the methods and figure legends, and was based on a 24hr bafilomycin treatment. The derived ratio of mCherry to GFP was calculated using FlowJo software. |

☒ Tick this box to confirm that a figure exemplifying the gating strategy is provided in the Supplementary Information.
